# Measuring What Matters for Children: A Systematic Review of Frequently Used Pediatric Generic PRO Instruments

**DOI:** 10.1007/s43441-021-00311-x

**Published:** 2021-06-17

**Authors:** Tasneem Arsiwala, Nuzhat Afroz, Kattayoun Kordy, Christel Naujoks, Francesco Patalano

**Affiliations:** 1 Novartis Pharma AG, Basel, Switzerland; 2 Novartis Healthcare Private Limited, Hyderabad, India; 3 Novartis Pharmaceuticals Corporation, East Hanover, NJ USA

**Keywords:** Adolescents, Children, ISPOR, Patient-reported outcomes, Pediatrics, Health-related quality of life

## Abstract

**Objective:**

To provide an assessment of the quality of the most frequently used self-reported, generic patient-reported outcome measures (PROMs) that measure health-related quality of life (HRQoL) in children against the good research practices recommended by ISPOR task force for the pediatric population.

**Method:**

Literature search was conducted on OvidSP database to identify the generic pediatric PROMs used in published clinical studies. The quality of PROMs used in more than ten clinical studies were descriptively evaluated against the ISPOR task force’s good research practices.

**Results:**

Six PROMs were evaluated, namely Pediatric Quality-of-Life inventory 4.0 (PedsQL), Child Health Questionnaire (CHQ), KIDSCREEN, KINDL, DISABKIDS and Child Health and Illness Profile (CHIP). All PROMs, except KIDSCREEN, had versions for different age ranges. Domains of physical, social, emotional health and school activities were common across all the instruments, while domains of family activities, parent relations, independence, and self-esteem were not present in all. Children’s input was sought during the development process of PROMs. Likert scales were used in all the instruments, supplemented with faces (smileys) in instruments for children under 8 years. KIDSCREEN and DISABKIDS were developed in a European collaboration project considering the cross-cultural impact during development.

**Conclusion:**

The comparison of the instruments highlights differences in the versions for different pediatric age groups. None of the PROMs fulfill all the good research practices recommended by the ISPOR task force. Further research is needed to define which age-appropriate domains are important for older children and adolescents.

**Supplementary Information:**

The online version contains supplementary material available at 10.1007/s43441-021-00311-x.

## Introduction

As with the adult population, establishing the health-related quality of life (HRQoL) for the pediatric population is essential. It is increasingly incorporated as outcome endpoints in pediatric clinical trials [1]. HRQoL is described as the impact of a disease or illness on a person's perceived physical, psychological, social, and emotional wellbeing [2, 3]. Patient-reported outcome measures (PROMs) are used in clinical trials to assess a child's functioning and evaluate health status improvement by medical intervention.

The patient-reported outcomes (PRO) Guidance to Industry, laid out by the U.S. Food and Drug Administration (FDA), supports the development of validated PROMs to collect PRO data. While the guidance is not specific to the pediatric population, there is a section on “special populations”, which outlines the challenges in developing pediatric instruments [4]. The guidance, however, does not mention methods for overcoming these challenges in the assessment of pediatric outcomes. The FDA PRO guidance states that for pediatric PROMs, the vocabulary, comprehension, and minimum age at which the children can provide valid and reliable responses must be considered. For the pediatric population, both self-reported and proxy-reported versions are frequently used. However, the FDA does not recommend proxy-reported outcomes but advocates self-reported outcomes whenever possible. Consistency between proxy-reported and child self-reports is low due to differences in perspectives, understanding, or in some cases, little insight into some concepts [5–7].

The International Society for Pharmacoeconomics and Outcomes Research (ISPOR) created a task force to address the challenges in developing and using pediatric PROMs. The outcome of this task force was a report by Matza et al. 2013 that recommends good practices for research and development of pediatric PRO instruments to support regulatory decision-making and medical product label claims [8]. Key good practices outlined in the report are to consider the developmental differences of the pediatric population and determine age-based criteria for the PRO administration, establish the content validity of the PROMs and child involvement, determine the need for informant-reported outcome instruments, ensure the instrument design and formatting is age-appropriate, and consider cross-cultural issues.

As no regulatory standards exist to define the appropriateness of pediatric PROMs, in our study, we utilized the good practices of the ISPOR task force to assess the quality of generic pediatric PROMs measuring HRQoL. As regulatory authorities favor self-reported measures, we evaluated child-reported versions of the instruments. PROMs used in more than ten studies were examined. The reliability and validity of the PROMs are not a focus in this assessment as several comprehensive publications have covered these aspects in detail [5, 9]. Since only self-reported instruments were evaluated, we focused mainly on whether the instruments were fit for purpose for that particular age group with respect to the developmental differences, content validity and concepts measured, design and format of the measure, and cross-cultural considerations.

## Methods

### Literature Search

A systematic literature review was conducted to identify generic PROMs used in pediatric clinical studies and clinical trials to measure health in children up to 18 years of age*. *The OvidSP database was searched for published literature, including clinical studies and clinical trials registered on clinicaltrials.gov until January 2021. The search terms used were “*patient-reported outcome”, “self-reported outcome”, “children”, “pediatric”, “clinical outcome assessment”, “health status”, “activities of daily living”, “life quality”, “quality of life”, “QoL” or “HRQoL”, “health-related quality of life”, “health status”, “questionnaire”. *We obtained 19,662 results screened as per the inclusion and exclusion criteria for generic PRO instruments. The results of the search were reviewed to identify eligible generic pediatric PROMs. Articles not related to pediatric PROMs, duplicates, or not published in English were excluded resulting in 1362 references. Pediatric PROMs assessing pain, fatigue, behavioral and psychological functioning were also excluded. Twenty-nine relevant generic PRO instruments were obtained. We subsequently focused only on the PROMs used in more than ten studies to ensure our search results were meaningful, further narrowing the list to the six PRO instruments. Only the self-reported of PROMs were assessed.

### Inclusion and Exclusion Criteria

Specific criteria were predefined to shortlist the eligible pediatric PROMs for review (Table 1).

**Table 1:** List of inclusion and exclusion criteria for selecting the generic pediatric health-related quality-of-life PROMs

*Inclusion criteria*	*Specifications*
Population	Children 0–18 years of age
Instrument type	Generic pediatric PRO instruments for self-report
Study type	Published clinical study
Language	English
*Exclusion criteria*	
Population	Non-pediatric population (ages > 18 years)
Instrument type	Preference based measures Instruments developed for children and adults Parent/proxy versions Disease or concept-specific instruments

### Data Extraction

**Fig. 1:**
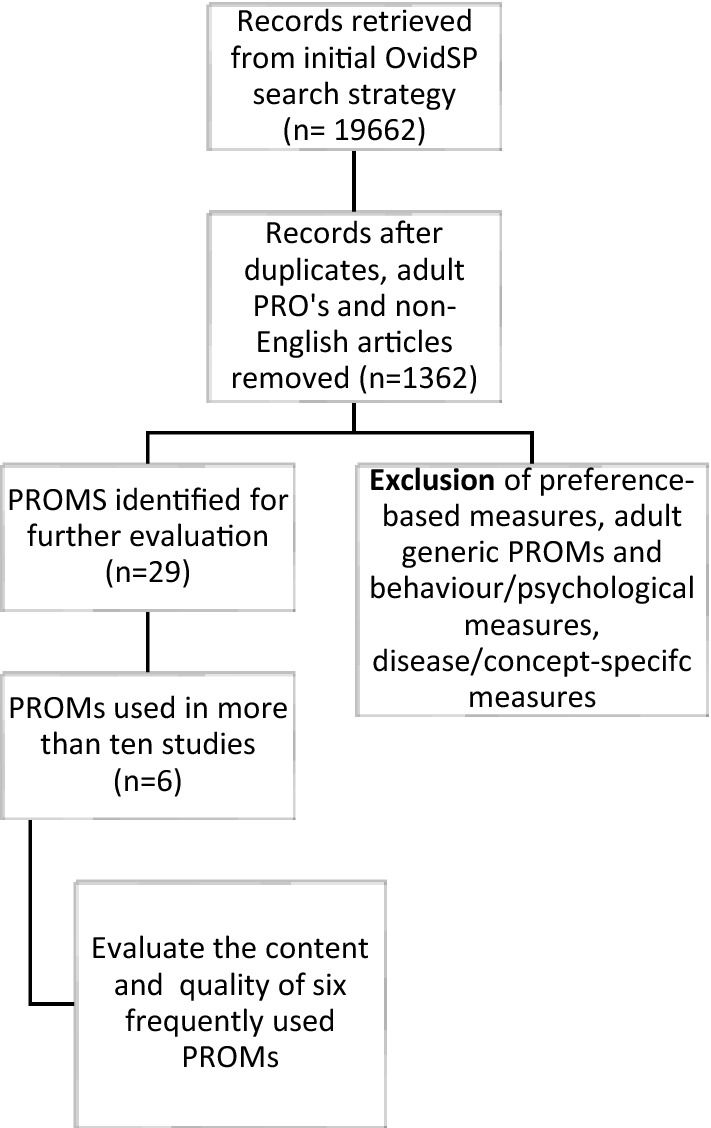
Flow chart showing the final selection of the eligible PRO measures (PROMs) for further evaluation

Six generic self-reported HRQoL pediatric PROMs from the literature search were identified for review. Next, using the names or the acronyms of the PROMs, an in-depth search to identify detailed information was conducted. Different self-report versions for the measures by age groups, the domains and item coverage of the PROMs, the content and item generation methods were compared. The aim was to determine whether respondents, i.e., children’s input was incorporated during the qualitative process, the design and format of the instrument, and cross-cultural considerations were evaluated. To help align on the ages on pediatric age groups, we define the pediatric age range as suggested by the ISPOR task force, describing young children (ages < 8 years), older children (ages 8–12 years), and adolescents (ages 13–18 years) (Fig. 1).

## Results

### Search Results

Twenty-nine pediatric PROMs met the inclusion criteria (Supplementary Table 1). Of the generic HRQoL measures identified, we evaluated the quality of PROMs used in more than ten clinical trials against the “Good Research Practices” recommended by Matza et al. (Supplementary Table 2). The six most frequently used generic PRO tools are Pediatric Quality-of-Life Inventory 4.0 (PedsQL), Child Health Questionnaire (CHQ), KIDSCREEN, KINDL-R, DISABKIDS and CHIP (Child Health Illness Profile). There are versions of these PROMs targeting specific age groups and different number of items (Table 2).

**Table 2:** Description of the characteristic of the six most frequently used generic pediatric instruments evaluated; details on the acronym and name, child-filled version of the instrument, age groups, method of item generation, response scales and domains

**Sr No**	**PROM**	**Child-filled instruments by age groups**	**Age**	**Content & item generation development process**	**Item generation with children**	**Pilot testing/validation with children**	**Response Scale**	**Recall period**	**Length of Instrument (Items)**	**Domains**	**References**
1	**Pediatric Quality-of-Life Inventory 4.0 (PedsQL)**	Young Child Report	5–7 years	NIA		✔	3-point Likert scales, with each response anchored to a happy to sad faces scale	1 month or 1 week	23; short- form- 15	Physical functioning, emotional functioning, Social functioning, School functioning	[13, 45]
		Teen Report Child Report	13–18 years 8–12 years	Extensive relevant literature research, open-ended interviews with patients (ages 8–18 years) and their families, and discussions with healthcare professionals such as pediatric specialists, nurses, and psychosocial specialists, followed by cognitive interviews with children. The wording and content of the child and teen report were as similar as possible, allowing for differences in developmental stages. The parallel items between the PedQL child and adolescent versions were used if the corrected item correlations was > 0 30	✔	✔	5-point Likert scale (never/ not at all to always)	1 month or 1 week			[13, 16]
2	**Child Health Questionnaire (CHQ)**	CHQ Child Form	5–18 years	Developed by the Child Health Project by review of literature and other instruments, validation studies	X	✔	4-to-6 point Likert scale	4 weeks; health change subscale- 1 year; global health, general health perception, family cohesion subscales- "in general"	87/ 45	Physical functioning, Role/social limitations—physical, General health perceptions, Bodily pain/ discomfort, Family activities, Role/social limitations, emotional/behavioral, Parent impact—time, Parent impact—emotion, Self-esteem, Mental health, Behavior, Family cohesion, Change in health	[14, 15, 21]
3	**KIDSCREEN**	KIDSCREEN	8–18 years	Literature review and a Delphi study were conducted to decide on structure and content (24 experts from 7 European countries). Feasibility of the items was determined by focus groups with children and their families	✔	✔	5-point Likert scale (never / not at all to always)	1 week	55/ 27/ 10	Physical Well-Being, Psychological Well-Being, Moods & Emotions, Self-Perception, Autonomy, Parent Relation & Home Life, Social Support & Peers, School Environment, Social Acceptance (Bullying), Financial Resources	[17, 32]
4	**KINDL**	Kiddy-KINDL	4–6 years	NIA			3-point rating-scale (never; sometimes; very often)	1 week	12	Physical, Emotional, Self-esteem, Family, Friends, School	[22, 46]
		Kid-KINDL Kiddo-KINDL	7–13 years 14–17 years	Kid and Kiddo-KINDL: The items were based on open-ended interview with children. The final questionnaire was constructed based on two pilot studies with 28 children each	✔	✔	5-point rating-scale (never; once a week; several times a week; every day and several times a day	1 week	24		[21, 22, 32]
5	**DISABKIDS**	DISABKIDS Smileys TAKE-6	4–7 years	Literature review, focus groups with children ages 4–7 years, and proxy focus groups (usually the mothers). Cognitive interviews were conducted for final item selection with a sample of 60 children with chronic medical conditions of ages 4–7 years	✔	✔	5-point Likert scale with smiley faces ranging from very sad to very happy		6	Mental (Independence, Emotion), Social (Inclusion and exclusion), Physical (Limitation, Treatment)	[43]
		DISABKIDS Chronic Generic Module (DCGM- 37) self-report	8–16 years	Literature research, focus group with children, and adolescents (ages 4–7, 8–12, 13–16 years) with mild to severe diseases conditions, caregivers and experts, cognitive interview with children and adolescents about relevance, and finally and expert review on item selection difficulty, and adequacy of items	✔	✔	5-grade response options ranging from 1 (never) to 5 (very often)	4 weeks	37; short form- 12		[19, 32]
6	**Child Health and Illness Profile (CHIP)**	Child Edition Child- Report Form (CHIP-CE/CRF)	6–11 years	The content, format, and wording were generated based on interviews with 114 elementary-aged children (ages 5–11 years), both healthy and chronically ill. Cognitive interviews and validation studies were done with children ages 8–11 years	✔	✔	4- or 5-point Likert scale	4 weeks	45	Satisfaction, Comfort, Risk avoidance, Resilience, Achievement	[35]
		CHIP-Adolescent Edition (CHIP-AE)	12–17 years	The content and items were generated based on literature reviews, focus groups with eight groups of 7 to 10 adolescents and two groups of 10 to 12 parents, health professionals, and expert panels. Cognitive interviews with healthy adolescents	✔	✔	4- or 5-point Likert scale	4 weeks	153	Satisfaction, Discomfort, Risks, Resilience, Achievement, Disorders	[44]

NIA denotes ‘no information available’

### Consideration of Developmental Differences and Age-Based Criteria for PRO Administration

The ISPOR task force paper recommends using different age ranges within the pediatric population that serve as a starting point to determine developmental differences and age-based content requirements for PRO assessment.

#### Age Ranges of the PROMs

Different versions of pediatric PROMs are available for different age ranges. The earliest age from which self-reported PROMs are available is age 4 years. Of the six PROMS evaluated, three (PedsQL young child report, Kiddy-KINDL, and DISABKIDS Smiley's measures) target ages < 8 years. PedsQL child report, Kid-KINDL, and CHIP-CE targeted children in the age group of 8–12 years. Instruments specifically for adolescents include the Kiddo-KINDL and CHIP-AE. CHQ, KIDSCREEN, and DISABKIDS chronic generic module-37 items (DCGM-37) have one instrument targeting a broad age range of 8–s18 years, with CHQ for 5–18 years. Thus, three instruments had separate questionnaires targeting particular subpopulations of pediatric age groups, while the other remaining instruments had a single questionnaire for a broad age group (Table 2).

#### Domain Comparison

The ISPOR task force recommends that pediatric instruments consider the target population's developmental stage and capture concepts relevant to the target population. Although dimensions such as physical or emotional wellbeing might be similar between various pediatric age groups, different dimensions of HRQoL must be recognized to reflect the target pediatric population's developmental age and social and emotional differences [10, 11].

#### Commonality of Domains Between Different Instruments

Physical functions, emotional functions, social relations, and school functions were included in all instruments. Although both CHIP versions don’t include physical functioning domain, they include items on physical discomfort and fitness activities under the domain of (dis)comfort and resilience. The instruments PedsQL, CHQ, KIDSCREEN, KINDL, DCGM-37, and CHIP include items on emotional functioning. In CHIP-AE, this is described under the discomfort domain. All PROMs include social functioning domain, which is relationships with friends and peers. DCGM-37 categorizes it further as social inclusion and social exclusion, while the CHIP instrument covers items of social functioning under the achievement domains. In the KIDSCREEN, it is covered under the domain of social support and peers. The school functioning domain includes items on school activities, interactions, and attention. It is included in PedsQL, CHQ (under domains of role/social limitations—physical and emotional/behavioral scale), KIDSCREEN, KINDL, and CHIP (achievement domain). DCGM-37 does not have school functioning domain but includes items on school functioning under the social inclusion domain.

#### Contrasting Domains Between the Different Instruments

We have attempted to focus on domains that potentially have an age-dependent impact on health assessment like family activities, parent relationships, independence, treatment, self-esteem, risk avoidance, financial resources, and social acceptance (bullying). For a complete list of domains in each PROM, refer to Table 2. *Family activities* domain includes items on perceived family support and the impact of illness on activities with the family and is included in CHQ and CHIP (in domain resilience). KINDL has the domain family, but the items target relationships with parents rather than family activities. KIDSCREEN and DCGM-37 don't mention items related to family activities. The domain of *parent relations* is vital for child development and wellbeing and is covered by CHQ, KIDSCREEN, KINDL (domain family), and CHIP (domain resilience). DCGM-37 and PedsQL don't address parent relationships. *Independence* is the ability to make decisions without any support [12]. KIDSCREEN and DCGM-37 include this domain. *Treatment* domain measures the impact of medication on daily activities and is included in DCGM-37. *Self-esteem* is the positive or negative attitude toward the self and assesses satisfaction with school, esthetic ability and looks, and perceived worth. It is mentioned in KINDL, CHQ, and CHIP (satisfaction domain). *Risk avoidance* measures the child's perception of how likely they are to participate in risky behaviors—both versions of CHIP measure this domain. Finally, KIDSCREEN includes domains of *financial resources* measuring the child's perceptions of their financial resources and *social acceptance* (bullying) with items on feeling rejected by peers in school.

### Content Validity and Item Generation

Children and adolescents can be effective content experts and share perspectives on their condition [12]. We did not find information on the methodology for content and item generation for the PedsQL young child report (ages 5–7 years) and the kiddy-KINDL (ages 4–6 years), but young children were involved in the studies for these instruments' psychometric validation [13]. The CHQ did not have pediatric input in the concept elicitation phase but involved children during pilot testing of the instrument [14, 15]. Items in the PedsQL and KINDL were created based on literature review, open-ended interviews with pediatric patients and their families and discussions with pediatric health care professionals (HCP) such as clinicians, nurses and psychosocial staff [16]. For item revision and field testing of the PedsQL, a new set of patients, families and HCPs were recruited, who underwent intense cognitive interviewing. KIDSREEN used the Delphi technique to establish the operationalization, questionnaire construction and content with input from a multidisciplinary group of experts consisting of psychologists, pediatricians, health services researchers and sociologists [17]. This was followed by focus groups with children to identify relevant dimensions and items and a multinational pilot testing with children [18]. Similarly, items in the DISABKIDS and CHIP were developed based on focus groups with children, parents and healthcare experts, followed by field testing to determine final domain structure and item feasibility [19].

### Child-Reported and Informant-Reported PROMs

PedsQL, CHQ, KIDSCREEN, KINDL-R, DISABKIDS and CHIP have both child-reported and proxy/parent versions of the instruments.

### Design and Format of the PROMs

The ISPOR task force recommends that the design and formatting of an instrument be age-appropriate for the target pediatric population [8]. This includes attention to the layout, font and using a large print for better clarity. Other formatting aspects include the overall health-related vocabulary, and reading level, response scales, recall period, pictorial representations, length of instrument, administration approaches and electronic data collection. The health-related vocabulary and the readability criteria are tackled at the item development and content validity stage to ensure an age-appropriate instrument.

#### Response Scales and the Use of Pictorial Representations

The response scales for PRO instruments are Likert scales with 3–5 points (a linear scale with numbers or words describing the range or severity of options, yes/no scales, and visual analog scale (a linear scale with numbers anchored at the two ends) [8, 20]. All six pediatric PROMS used the Likert scale. Instruments for children less than 8 years use a simplified Likert scale supported with facial expressions or illustrations as seen in the PedsQL young child report. DISABKIDS TAKE-6 instrument has a 5-point Likert scale anchored with facial expressions (smiley faces) to facilitate responses. CHIP-CE uses two illustrations for each question with five possible response circles, which gradually increase in size to indicate increasing frequency or amount.

#### Recall Period

The ISPOR task force recommends that a shorter recall period of 24 h is more favorable for regulatory purposes. CHIP, DISABKIDS, CHQ, and PedsQL have a recall period of four weeks or one month. PedsQL also has an acute version with a recall period of one week. KIDSCREEN and KINDL have a one-week recall period.

#### Length of Instrument

The ISPOR task force recommends carefully considering pediatric PROM's length and avoiding long questionnaires for younger children [8]. A comparison of the number of items across versions of the instruments revealed more items in the versions of the PROMs for older children and adolescents. The adolescent version CHIP-AE has the highest number of items, 153. CHIP-AE has 71% more items compared to CHIP-CE, the young children version. The DCGM-37 for ages 8–18 years has 37 items, while DISABKIDS-Smileys TAKE-6 for ages 4–7 years has 6 items. The difference between the items of the two age versions is 84%. The difference in the number of items for the Kiddy- and Kid/Kiddo-KINDL is 50%, while PedsQL has the same number of items for young children, children and adolescent forms.

#### Administration Approach

According to PedsQL guidelines, older children and adolescents may independently fill the PROM after introductory instructions from the administrator. Similarly, children can complete the CHQ, DCGM-37, CHIP-AE and KINDL-R (Kid-KINDL and Kiddo-KINDL) independently [21, 22]. For young children whose reading skills are not advanced to the necessary standard, questionnaires can be administered in interview form (face-to-face or by telephone), like for Kiddy-KINDL and CHIP-CE [21, 22]. The PedsQL administration guidelines recommend that for the Young Child Report, an administrator reads all the instructions and each item and repeats the recall period at the start of each subscale. The KIDSCREEN instrument can be administered independently at home, in a classroom or other settings, or by interview method via telephone or face-to-face.

#### Electronic Data Collection

PedsQL, CHQ have ePRO versions available. Likewise, the KINDL-R, KIDSCREEN, DCGM-37 and CHIP have a computer-assisted version for children and adolescents.

### Cross-Cultural Issues

As PROMs are designed for widespread use, language and cultural differences can impact their acceptance and use. For this reason, two European projects were initiated, resulting in the KIDSCREEN and the DISABKIDS measures. As these instruments were created as a European collaboration with input from children, families, healthcare providers, and subject matter experts across multiple European countries, cross-cultural influences and language translations were considered from the start.

## Discussion

Pediatric PROMs are commonly used in pediatric clinical studies to monitor outcomes in children [23]. The ISPOR task force has defined good research practices to characterize the appropriateness of pediatric PROMs. Previous studies have demonstrated the appropriateness of PROMs using psychometric properties as a benchmark but lacking focus on the other good practices. To this end, we have assessed the quality of generic pediatric PROMs, focusing on these good research practices recommended by the ISPOR task force and not the instrument's psychometric properties. Furthermore, we identify the shortcomings of the frequently used generic PROMs and highlight which aspects need to be strengthened.

Using a literature search we identified the six most frequently used generic PROMs in pediatric clinical studies. The method of item generation, differences in domains, formatting and design considerations, cultural variations of the six PROMs were assessed against the good research practices of the ISPOR task force report. While we focus on generic PROMs that have items relevant to any disease indication, disease-specific PROMS with items targeting the symptoms, and feelings of a particular disease are also available.

The ISPOR task force recommends to carefully consider the developmental differences across different pediatric age ranges. One way to achieve this is to have multiple age group-specific versions of the instrument, where the item structure, wording and domains are optimized for the pediatric subpopulation. We identified that all PROMs, except KIDSCREEN, had different age group versions, varying in wording, length of questionnaire, formatting and concepts.

For an instrument to be age-appropriate for the users, it must capture relevant domains. A child’s social and emotional experiences are critical for overall personal development [24]. Unlike adult HRQoL PROMs that commonly assess physical health, emotional or mental health, and social wellbeing, these domains are insufficient to capture a child’s overall wellbeing. There is a bidirectional influence between children and their multiple social contexts, i.e., children actively exert influence on their social context while being simultaneously shaped by these contexts [25]. These social contexts may include families, peer relationships or school activities. For example, children’s family activities are linked to their behavior, social recognition, friendships, and acceptance from peers [26, 27]. Other concepts like parent relations, independence (autonomy), extracurricular activities, impact of treatment, and self-esteem might also be important indicators toward the wellbeing of children and adolescents [28, 29]. Hence, the ISPOR task force recommends that a child’s social and developmental contexts be considered and be incorporated into pediatric PROMs [8, 30]. Thus, a pediatric instrument's age and concept appropriateness are examined during the concept elicitation and debriefing phases.

We observed that although all the PROMs evaluated physical, emotional, social and school functioning domains, none covered all the additional domains mentioned above. This is expected because each pediatric PROM was developed by its own goal and unique qualitative methodology by engaging with different children, both healthy and those with multiple illnesses, caregivers and pediatric health experts [31]. For example, the purpose of the KIDSCREEN project was to contribute to health reporting, while DISABKIDS project was to contribute to improving disease-related research in clinical studies with chronically ill children (DCGM-37) and has additional disease-specific modules for certain diseases [32].

Research has shown that children can provide a unique perspective and be effective content experts [12]. Hence, during PROM development and item generation stage, the relevant concepts for children of different ages is obtained by the concept elicitation process done by focus groups and interviews [33]. Like the FDA PRO guidance, the ISPOR pediatric PROM assessment task force recommends incorporating responder input in developmental phases [4]. All PROMs, except CHQ, incorporated children’s (respondents) input during the developmental phases by concept elicitation method, while all six PROMS involved children in the pilot testing of the PROMs.

The design and formatting of PROMs are important to ensure easier comprehension. The layout, font type, font size, length of an instrument must be considered. The readability also impacts PROM usage and is tested in the cognitive debriefing phase. However, readability is not necessarily assessed going into trials. Examining readability should be a more common practice in instrument development and administration [34]. The number of items must be taken into account while developing pediatric instruments. This improves the attention and comprehension of younger children who have limited attention span, who may otherwise exclude items or not answer carefully, resulting in erroneous or less reliable data [8, 35]. Compatible with this recommendation, the child and adolescent versions of PedsQL, KINDL, DISABKIDS, and CHIP are longer with more items than the young children's versions. The recall period is another important criterion in pediatric PROMs. The optimal duration of a recall period depends on the child’s understanding of elapsed time and memory. The choice of recall period depends on the purpose of measurement and the frequency and intensity of the concept analyzed. ISPOR recommends a short recall period of 24 h for PROMs for regulatory decision-making [8]. None of the PROMs we evaluated have a 24 h recall period. KINDL, KIDSCREEN, and PedsQL acute versions have a recall period of one week. CHIP, CHQ, PedsQL, and DCGM-37 have a recall period of four weeks. While a short recall period is favored, the downsides include frequent measurements, respondent burden, and failure to capture symptoms outside the recall period window [8]. The recall periods in these PROMs are an attempt for a practical solution. However, if used in a clinical study, the rationale should be justified based on the situation. If a 24 h recall period is necessary depending on the purpose of the study or regulatory feedback, the current PROMs can be adapted and validated for daily measurements. Alternatively, further research could be done to develop patient diaries with once or twice daily response options to overcome the challenges of the long recall period.

The instrument response scales are important to the overall relevance and quality of the pediatric instrument. The Likert scale is among the most common response measurements [36]. The Likert scale responses typically vary from 3-to-5 points. Studies have shown that children understand and can grade 5-Likert scales adequately, especially for concrete tasks, but the items and response options need to be worded carefully [36]. To enhance judgment of response, in tests for young children under 8 years, a simplified 3-point Likert scale is supplemented with faces (smileys) or cartoon illustrations to facilitate comprehension. Although there is limited evidence on the advantage of pictorial illustrations in PRO instruments, a study showed they keep the child engaged and thus help in faster completion of the items [37].

Similarly, PROM administration procedures depend on the age group of the respondents. As per the task force report, older children and adolescents can be expected to complete the PROM independently. Generally, the comprehension and reading ability of older children and adolescents favors independent PROM completion with little to no support. However, an interview approach with the questions read to children is advised and implemented for younger children or children with learning disabilities. All the six PROMs take this administration approach into account. While the ISPOR guidance supports self-reported measures for children, proxy-reported outcome measures may be used in children who cannot complete the PROMs by themselves due to developmental or cognitive challenges, illnesses, and are too young. It is also recommended by ISPOR task force to implement electronic PRO data collection [8]. Many children are familiar with digital screen-based activities and may prefer electronic means of collecting PRO data. The advantages are improved efficiency and fewer errors or omitted responses [38]. All six pediatric PROMs had electronic versions for data collection.

Variations in cultural perspectives can lead to erroneous conclusions as well. Differences in education systems and the subsequent impact on language and reading abilities, willingness to talk about specific topics, preference to be interviewed in parents' presence, or be independent responders can all be sources of cultural biases. These aspects are addressed when the tool is developed during the content validation phase. We identified that DISABKIDS and KIDSCREEN projects were a step in the right direction, where cross-cultural effects and multiple translations of PRO instruments were considered right from the beginning in a European collaboration. During large clinical studies, with cultural variations in perspective, the interpretation of the PRO tools is acknowledged as a limitation if not validated in the specific ethnicity. When a PROM is translated into another language, the translation and cross-cultural adaptation process should follow the best-practice methodology [39].

This study shows that there are differences among the pediatric PROMs and the heterogeneity in the pediatric population, thus making the selection of a PROM for a clinical trial challenging. The accurate measurement of PRO endpoints relies on opting for a suitable PROM applied to the correct population. Selecting a suitable PROM depends on the disease indication, purpose and duration of the study, responder population, frequency of measurements, concepts/primary domains of interest, administration approach, and psychometric properties of the PROM. An extensive literature search of existing generic and disease-specific PROMs, their use in clinical studies, along with qualitative interviews with KOL/experts and caregivers are performed to facilitate the selection of suitable PROMs for trials. Additionally, the COSMIN checklist can aid in the evaluation of the measurement properties of a PROM to ensure scientifically appropriate conclusions of the concepts measured. In a trial with multiple age groups that do not align with the age range of the PROMs, one might adapt the existing validated PROMs for the age groups in the trials. Also, it is important to use validated measurements that are appropriate for the age group, and hence combining different validated PRO tools in one trial might be an option.

## Future Implications for Practice and Research

To overcome the limitations of the current pediatric instruments, pediatric PROMs with computerized-adaptive test (CAT) software are being favored in children. The CAT versions of instruments use software algorithms to select optimal items based on the respondent’s overall patterns of responses [40]. Such PROMs address problems associated with readability and age-appropriate concepts by customizing the items to the respondent level based on prior responses. Also, there’s potential to explore digital or game-like apps to make pediatric self-measurements more engaging for children. Apps like electronic Pain Assessment Tool (ePAT) or PainChek® used in older adult patients, use AI technology for facial analysis to evaluate the presence and intensity of pain [41, 42]. Such digital apps and game-like formats for PRO assessments will help children be more interested in providing measurements and improve the evaluation of symptoms that children struggle to verbalize or articulate.

While it is important to have new PROMs using the digital platform, this study has shown fundamental differences and similarities exist between the PROMs. Unlike psychometric properties, similar focus is required for other aspects of the PROMs mentioned in the ISPOR task force. While we have compared the most used PROMs along these aspects, further work is needed to translate these findings into consistent new PROMs. Establishing consistency between the PROM instruments is pertinent to improving patient outcomes in the pediatric population.

## Conclusion

We identified six PROMs widely used in studies for children to reliably report their health states. Based on our work and the good practices and issues identified by the ISPOR task force to develop PROMs for children, we believe that many areas of pediatric PROMs need further research. An instrument that appropriately captures all relevant age-appropriate domains in PROMs to measure outcomes in pediatric drug development is yet to be developed. Determining appropriate concepts is complicated as the pediatric subpopulations are developmentally heterogeneous [8]. More studies are required to agree on dimensions necessary for children of different age groups, especially from childhood to adolescence and adolescence to adulthood. We believe research is needed to understand the optimal length and number of items of pediatric PROMs, being mindful that in addition to children being easily distracted, many of the children are not entirely healthy and cannot be encumbered further. Additional methods like exit interviews with children and caregivers at the end of a trial on their experience filling PROMs could give valuable insight into optimal PROM length. Such research will expand the toolbox of fit-for-purpose pediatric PROMs overcoming the gaps in the current PROMs and are suitable for regulatory decision-making purposes.

## Supplementary Information

Below is the link to the electronic supplementary material.

Supplementary file 1 (DOCX 19 kb)
