# Supplementary material for: Measuring What Matters for Children: A Systematic Review of Frequently Used Pediatric Generic PRO Instruments
Source: Ther Innov Regul Sci. 2021 Jun 17;55(5):1082–95. doi: 10.1007/s43441-021-00311-x (PMC8332594; doi:10.1007/s43441-021-00311-x)
Supplement: Supplementary file 1 — (DOCX 19 kb) [file 43441_2021_311_MOESM1_ESM.docx]

**Supplementary Tables:**

Supplementary Table 1

*Complete list of generic PROMs identified from the OvidSP literature search used in all published clinical studies.*

| **No.** | **PROM instrument** | **No. of publications** |
| --- | --- | --- |
| 1 | Paediatric Quality-of-Life Inventory 4.0 (PedsQL) | 257 |
| 2 | KIDSCREEN | 43 |
| 3 | Child Health Questionnaire (CHQ) | 33 |
| 4 | KINDL | 25 |
| 5 | DISABKIDS | 23 |
| 6 | Child Health and Illness Profile - Adolescent Edition (CHIP) | 13 |
| 7 | TNO-AZL Children Quality-Of-Life Questionnaires (TACQOL) | 10 |
| 8 | Childhood Health Assessment questionnaire (CHAQ) | 7 |
| 9 | French Vecú Santé Perçue de l'Adolescent (VSP-A) | 5 |
| 10 | Autoquestionnaire Qualite de Vie Enfant Image (AUQUEI) | 3 |
| 11 | Infant Toddler Quality of Life Questionnaire (ITQOL) | 3 |
| 12 | Youth Quality of Life Instrument (YQOL) | 3 |
| 13 | World Health Organization Quality of Life BREF | 3 |
| 14 | Child Quality of Life Questionnaire (CQoL) | 2 |
| 15 | Generic Children's QoL Measure (GCQ) | 2 |
| 16 | Quality of My Life (QoML) questionnaire | 2 |
| 17 | Infant QoL Instrument (IQI) | 2 |
| 18 | Berner Questionnaire for Well-Being (BFW) | 1 |
| 19 | Comprehensive Health Status Classification System – Preschool (CHSCS-PS) | 1 |
| 20 | Dartmouth Primary Care Cooperative Information Project Charts | 1 |
| 21 | Exeter QOL measure | 1 |
| 22 | Gothenburg Quality of Life | 1 |
| 23 | Infant health-related Quality of life Instrument (IQI) | 1 |
| 24 | PedsQL Infant Scales | 1 |
| 25 | Vécu et Santé Perçue de l’Enfant for children aged 12–17 y | 1 |
| 26 | Warwick Child Health and Morbidity Profile | 1 |
| 27 | Nordic QOL Questionnaire | 1 |
| 28 | Quality of Life Profile Adolescent Version (QOLPAV) | 1 |
| 29 | Kids-CAT, a computer-adaptive test (CAT) | 1 |

Supplementary Table 2:

*List of the good practices for the assessment of children and adolescents as discussed in the ISPOR task force report (*Matza LS, Patrick DL, Riley AW, Alexander JJ, Rajmil L, Pleil AM, et al. Pediatric Patient-Reported Outcome Instruments for Research to Support Medical Product Labeling: Report of the ISPOR PRO Good Research Practices for the Assessment of Children and Adolescents Task Force. Value in Health. 2013;16(4):461-79*).*

| **Good Research Practice** |
| --- |
| 1. Consider Developmental differences and age-based criteria for PRO administration. |
| 1. Establish content validity of pediatric PRO instruments. |
| 1. Determine Whether informant-reported outcomes measure are necessary. |
| 1. Ensure the instrument is designed and formatted appropriately for the target age group. |
| 1. Consider Cross-cultural issues |
